# Psychological factors in exceptional, extreme and torturous environments

**DOI:** 10.1186/s13728-016-0048-y

**Published:** 2016-06-01

**Authors:** John Leach

**Affiliations:** Extreme Environmental Medicine & Science Group, Extreme Environments Laboratory, University of Portsmouth, Portsmouth, PO1 2ER UK

**Keywords:** Exceptional environment, Extreme environment, Psychological torture, Sensory duress, Physical stress, Degradation, Pathogenic, Salutogenic

## Abstract

Our cognitive system has adapted to support goal-directed behaviour within a normal environment. An abnormal environment is one to which we are not optimally adapted but can accommodate through the development of coping strategies. These abnormal environments can be ‘exceptional’, e.g., polar base, space station, submarine, prison, intensive care unit, isolation ward etc.; ‘extreme’, marked by more intense environmental stimuli and a real or perceived lack of control over the situation, e.g., surviving at sea in a life-raft, harsh prison camp etc.; or ‘tortuous’, when specific environmental stimuli are used deliberately against a person in an attempt to undermine his will or resistance. The main factors in an abnormal environment are: psychological (isolation, sensory deprivation, sensory overload, sleep deprivation, temporal disorientation); psychophysiological (thermal, stress positions), and psychosocial (cultural humiliation, sexual degradation). Each single factor may not be considered tortuous, however, if deliberately structured into a systemic cluster may constitute torture under legal definition. The individual experience of extremis can be pathogenic or salutogenic and attempts are being made to capitalise on these positive experiences whilst ameliorating the more negative aspects of living in an abnormal environment.

## Background

Human beings are highly resistant and adaptable to the most varied environmental conditions [1]. Our cognitive system is mostly reliable, robust and flexible in interacting with different environments and its underpinning of our behaviour has been key in enabling the human species to inhabit every type of terrain on the planet.

We have adapted to operate within an optimal environment that means both an interaction with that environment through goal-directed behaviour, and possessing some control and choice over that environment; however, the more the environment deviates from the optimal the less control we have and the more reactive we become to it. Nonetheless, any person can be considered an active agent, capable of adapting and coping, not only in normal environments but also in exceptional, extreme and even torturous environments.

Some environments or situations can be considered as ‘exceptional’ in that, whilst we have not naturally adapted to these environments, coping behaviours can be learned to enable us to exist within them. These environments are sought out by certain types of people, such as explorers and adventurers, submariners, divers working under sea (who may also be isolated for extended periods in hyperbaric saturation chambers), astronauts involved in space missions and working in space stations, and even certain religious sects whose practice involves withdrawal from interactions with the outside world even to the pursuit of a solitary life. Whilst some people choose to spend part of their lives in such exceptional environments, others find themselves unwittingly consigned to such environments including prisons, intensive care units (ICUs), hospital isolation wards and suchlike.

Any deviation from the optimal, particularly if personal control is lost, can result in stress. This stress can be *dis*tress or pathogenic stress if psychological and psychophysiological dysfunction occurs. Alternatively, stress can be *eu*stress or salutogenic stress which has health enhancing effects and positive outcomes for the individual [2]. The positive psychological effects of living in abnormal environments or through experiencing abnormal situations have recently been identified in such areas as polar research, space exploration and returnees from captivity [3, 4].

Attempts are made to ameliorate the duress of living in exceptional environments by such methods as engineering the physical design, regulating temporal and photopic cycles, providing accessible communication channels to the outside world etc. Those living in these environments may also reduce the duress of their situation through developing coping skills that can serve them in either adapting to, or in ignoring, the environment [2].

An ‘extreme’ environment can be considered one in which the environmental stimuli are of such an intensity that they have a dysfunctional impact on an individual’s personality or psychological integrity. The person in such an environment has little or no control over the situation in which he or she finds themselves. Furthermore, those who do have control over the environment, instead of attempting to ameliorate the duress may deliberately intensify the conditions to increase the pain and psychological trauma being experienced. The further extension of an extreme environment, coupled with the deliberate infliction of psychological duress that exceeds a person’s cognitive tolerance, may induce psychological trauma and can be considered torture.

That an extreme environment or experience can be ‘tortuous’ is worth considering further. Torture is a legal construct that has so far eluded a single operational definition. Whilst Article 3 of the European Convention on Human Rights states that, ‘No one shall be subjected to torture or to inhuman or degrading treatment or punishment’, the European Court of Human Rights (ECHR) chose not to define torture and refers to Article 3 as a living instrument [5] meaning that what constitutes ‘torture’ is expected to change with time.

As an instance of such change, the author attended school in England in the days when corporal punishment (slapping or striking usually with a hand, ruler, plimsoll or cane) and often administered publicly, was commonly accepted as allowable practice even by the recipients. It was a time of casual but regulated brutality. Indeed, in 1993 the ECHR held that giving a 7-year-old boy three ‘whacks’ with a gym shoe over his trousers was not a forbidden or degrading treatment [6]. It was in 1999 that corporal punishment was banned in English schools as constituting inhuman and degrading punishment.

What may be termed psychological punishment was also regularly practiced: being made to stand in the corner of a room facing the wall (stimulus reduction), sometimes for long periods (stress position), being sent to sit in a junior class (social humiliation), having to write out ‘lines’ five-hundred times (monotony), and I recall, one particular master who would inform the selected pupil at the end of school on a Friday afternoon that he would cane him first thing Monday morning (instilling fear and anxiety).

The elements of torture, inhuman and degrading treatment are deliberately distinctive. Behaviour that is inhuman will be degrading but may not constitute torture, whereas torture will comprise behaviour that is both inhuman and degrading. Torture is a severe form of inhuman treatment, but there is no objective element of distinction between the two categories: ‘*Torture is not an act in itself, or specific type of acts, but it is the legal qualification of an event or behaviour, based on the comprehensive assessment of this event or behaviour*’ [7].

Lord Parker of Waddington expressed succinctly the problem in defining psychological torture in his report to Parliament following his inquiry into the alleged torture of IRA suspects by British Forces in 1971: ‘Where does hardship and discomfort end and, for instance, humiliating treatment begin, and where does the latter end and torture begin?’ The answer he concludes rests on ‘words of definition’ and consequently opinions will differ. This differing of opinion over what constitutes ‘psychological torture’ is a recurring problem. It has been suggested that torture is a matter of degree, and can be defined by the amount of mental suffering involved, but as a former US surgeon-general asked somewhat ironically, ‘How loud does a scream have to be?’ [8]. However, this difficulty with providing an objective assessment of psychological torture has been recognised: Sir Nigel Rodley, former UN Special Rapporteur on Torture states, ‘[T]he notion of “intensity of suffering” is not susceptible of precise gradation, and in the case of mainly mental as opposed to physical suffering, there may be an aura of uncertainty as to how… [to assess] the matter in any individual case’ [9].

Torture comprises three main elements [5]: (i) the infliction of severe mental or physical pain or suffering; (ii) the intentional or deliberate infliction of the pain; (iii) the pursuit of a specific purpose, such as gaining information, punishment or intimidation.

Torture is also characterised as having deliberate intent; in other words, torture is cruel and inhuman treatment that is delivered with a purpose. The infliction of inhuman and cruel acts without intention does not constitute torture. This is derived from the principle established by the former Attorney General for England and Wales Sir Edward Coke (1552–1634) that, *actus non facit reum nisi mens sit rea* (an act does not make a person guilty unless his mind is also guilty). However, it has been argued that the intention of the perpetrators of psychological torture is not necessary and an ‘extensional’ definition has been proposed in which a delineated set of practices can be defined as constituting psychological torture [10]. These practices include isolation, sleep deprivation, confinement, especially in small places, sensory deprivation or overstimulation, indefinite detention etc. For the purpose of this paper it is worth revisiting the Royal Air Force combat survival training manual [11] that refers to the application of psychological torture against a person as an ‘assault on the mind’. This definition is both simple and effective.

Both physical and psychological duress may cause pain and suffering but only physical trauma can leave overt marks on the body. In exceptional environments these marks can result from frostbite, decompression sickness, intubations, biopsies etc. In tortuous environments these can occur through beatings, suspensions, burnings etc. Consequently, psychological torture has often been considered by its practitioners as ‘torture-light’ or ‘no-touch torture’; yet whilst physical and psychological torture can be separated they cannot be divorced and it has been pointed out that psychological torture still requires extensive physical manipulation (e.g., physical confinement, keeping people awake) and, therefore, psychological torture is also an assault on the body. Furthermore, both physical and psychological torture compromise the mind–body integrity and produce physical and functional changes in the brain that can be identified through neuropsychological testing and neuroimaging [12, 13].

Assaults on the mind can be divided broadly into three categories: psychological (isolation, sensory deprivation, sensory overload, sleep deprivation, temporal disorientation); psychophysiological (thermal, stress positions), and psychosocial (cultural humiliation, sexual degradation). These will be considered in turn.

## Psychological

### Isolation

Isolation involves both the restriction of environmental stimuli and the reduction in the quantity and the quality of stimuli that are psychologically and socially meaningful. Often it is the reduction in social interaction that is the more problematic.

As but one example, Captain Howard Rutledge an American prisoner of war who was held captive by the North Vietnamese stated that he was constantly amazed at how isolation, ‘…could break my own willingness to resist. Physical torture may have ended, but there is still no torture worse than years of solitary confinement’ [14]. Rutledge continues:

‘It’s hard to describe what solitary confinement can do to unnerve and defeat a man. You quickly tire of standing up or sitting down, sleeping or being awake. There are no books, no paper or pencils, no magazines or newspapers. The only colo[u]rs you see are drab gr[e]y and dirty brown. Months or years may go by when you don’t see the sunrise or the moon, green grass or flowers. You are locked in, alone and silent in your filthy little cell breathing stale, rotten air and trying to keep your sanity’.

Rutledge’s last point is important; probably more than any other environmental factor solitary confinement is the one most likely to induce psychiatric disturbance. There is a real fear of psychological and personal deterioration and transformation into a ‘non-being’ and those unable to adapt to isolation tend to become psychotic [15, 16].

As human beings we rely on social contact to inform our perception of the world, the environment, ourselves and of reality. Social connectedness is a pre-requisite to long-term social adjustment and a lack of social contact makes it difficult to distinguish what is real from what is not or what is external to what is internal [17]. We often assess and maintain our own personality and sense of self and identity by seeing how we are reflected in the behaviour and responses towards us of other people, a process termed in 1902 the ‘looking glass self’ [18]. Social cognition would also suggest that our minds are in part comprised from other minds; consequently, isolation from other people can lead to cognitive dysfunction, mental withdrawal and in some cases complete psychological dissolution [19].

There is a distinction between solitude and isolation. Solitude usually implies a temporary state of being alone that may be deliberately sought and often brings with it psychological replenishment. Isolation can be a consequence of entering an exceptional environment (e.g., overwintering on a polar station) or can be imposed involuntarily either through misadventure (e.g., shipwreck) or through intent such as punishment or being taken hostage.

In isolated posts in the Arctic and Antarctic a psychological decline has been observed which is referred to variously as a ‘winter-over syndrome’, Polar T3 syndrome or subsyndromal seasonal affective disorder. The condition is characterised by an increasing depression, irritability, impairment in cognitive functioning, social withdrawal, a degradation in relationships coupled with an increase in interpersonal hostility and a lowering of the threshold for triggering anger, sleep disturbance, loss of appetite, anxiety and apathy. These symptoms can arise even after investment in the polar station structure and environment (for a review see [1]). Whilst many studies into polar exploration tend to report the more pathogenic effects, particularly of long periods of isolation in confined groups, evidence suggests significant individual differences in tolerance to this condition [20] and the symptoms reported reach clinical levels only in very few instances [21]. Similarly, astronauts are also reported to show fatigue, irritability, emotional lability, attention and concentration difficulties, restlessness, heightened perceptual sensitivities and sleep disturbances (for a review see [3]). But, some people do report actual positive benefits from the polar experience [22] and from space missions [23] (see below).

In other exceptional settings the literature continues to find adverse psychological effects arising in hospital patients placed in isolation wards including anxiety, irritability, apathy, aggression, difficultly in concentration, hallucinations and psychotic reactions in some individuals [24] most of which arise from isolation itself (for a review see [25]). However, a study into short-term hospital isolation (median length = 4 days) found no significant psychological impact [26].

Polar stations, space missions and isolation wards can be considered exceptional environments whereas enforced isolation in solitary confinement is an extreme environment. Solitary confinement is linked historically with abuse and is often considered by its victims to be used deliberately to break a person’s will and to induce insanity. The question of sanity is interesting as it has been suggested that ‘madness’ amongst people in isolation may, in part, be a functional and adaptive response to the environment [27].

Solitary confinement is known to be effective in disrupting a person’s psychological equilibrium, which is the reason for its widespread use as a form of psychological assault a key purpose of which is to weaken a person’s will to resist and to make his or her personality more malleable. As early as 1910 the Washington Supreme Court observed that confessions, both true and false, are more easily extracted from prisoners who are subjected to solitary confinement [16].

Enforced isolation was not always perceived to be harmful; in fact, positive benefits have been claimed for the isolated life of a monastic recluse. However, these are volunteers for a life of withdrawal to an exceptional existence and such a life of ‘perfect desolation’ is not the one chosen by most people [28]. There are very few robust experimental studies due to methodological difficulties although there was a flurry of interest in the effects of isolation during the 1960s and 1970s.

Complete isolation degrades cognitive function and coupled with social isolation can destroy the personality [29]. The effects are difficult, although not impossible, to resist and rapidly produce psychopathology even in healthy people. Almost every study of non-voluntary isolation has found negative psychological effects from confinement that lasted 10 days or more consistent with general signs of maladjustment [30]. The initial response to isolation is a natural anxiety plus introspection, a concern with the past and a direct engagement with the immediate environment followed by bursts of restlessness, pacing up and down, yelling and banging followed by sleep disturbance, difficultly in maintaining attention, daydreaming, a tendency to withdrawal, dissociation from the situation and a physical and psychological regression. This period of anxiety and adjustment to the isolation routine lasts from 1 to 3 weeks. Further isolation of 4–6 weeks leads to a feeling of dejection and increasing dependency, loss of initiative and spontaneous activity, lack of interest in personal appearance, immobility and vacant gaze. The process suggests that inactivity and lack of social contact leads to a loss of meaning which in turn leads to despair. The victim then focuses on this despair, which becomes self-perpetuating. It is this loss of meaning that seems to trigger a failure to adapt to an isolated environment. The initial onset of severe apathy and lethargy leads to an inability to concentrate, to think cogently and to initiate behaviour suggesting impairment in executive function [31].

It has been suggested that solitary confinement can cause a specific psychiatric syndrome [15] although this is still under debate. Nonetheless, there is little doubt that some victims will exhibit all or most of these symptoms, some will show a few symptoms and a few will show no visible symptoms depending on the pre-morbid adjustment of the individual and the context, length and conditions of confinement [32].

Psychological disturbance can occur after only a few days of isolation, however, recovery can also be rapid, at least in those who are psychologically robust prior to entering isolation but long-term effects may be observed in those who had psychological or emotional problems beforehand or whose isolated environment was particularly brutal. When isolated individuals attempt to re-enter the normal world the perceptual distortions they experienced when cut-off from sensory stimulation cause difficulties but these expressions are underpinned by more subtle psychological, emotional and cognitive disturbances [33]. One commonly reported effect is the difficulty those removed from long-term isolation have in speaking. There are suggestions that solitary confinement can leave residual psychological problems and the possibility of a chronic post-isolation syndrome existing has been debated [32].

### Sensory deprivation

Isolation, solitary confinement and sensory deprivation lie along a spectrum of diminishing richness of physical and social stimuli. Although not admitted at first, funding for research into sensory deprivation was given to study the putative effects of ‘brain washing’ following concern over Chinese and North Korean interrogation methods and the forced confessions presented at Communist show trials during the Soviet era. It was believed that techniques of sensory and perceptual deprivation were instrumental in reducing resistance to interrogation, heightening suggestibility and particularly enabling attitude change and indoctrination where it was feared that a person could lose the ability to control his own thoughts and be reduced to repeating the thoughts that have been implanted from outside. Certainly, experiments in sensory deprivation showed that subjects deprived of visual, auditory and tactile stimulation for up to seven days developed increased suggestibility [34].

Sensory deprivation studies flourished during the 1950s and 60s and were later applied to understanding the problems reported amongst people living in naturally isolated and sensorily deprived environments such as overwintering polar expeditions, long endurance submarine operations and space missions. However, sensory deprivation studies decreased markedly from the mid-1970s onwards which may be partly due to the discovery of heavy military funding for the programmes and partly to no new major findings being discovered [34].

The initial academic studies of sensory deprivation are attributed to the Canadian psychologist Donald O. Hebb (1904–1985). Hebb isolated volunteers in cubicles and either reduced sensory input to a minimum level (sensory deprivation) or presented subjects with unpatterned stimuli at a constant level (perceptual deprivation). After 4 h many subjects found difficulty in rational thinking and after 48 h most reported hallucinating. In a related study seventeen healthy volunteers were placed in a tank type respirator used by polio patients that reduced tactile stimulation, constrained movement and was combined with low light levels. Five subjects completed the full 36 h duration of the experiment with all reporting feelings of anxiety and with half reporting vivid distorted imagery, hallucinations, altered mood states and time distortions [35]. Other symptoms repeatedly reported in sensory and perceptual deprivation research include cognitive impairment, concentration problems and impaired memory, distressing mood states and psychotic behaviour [16, 36]. Further reduction in patterned stimuli was produced through submersion in water tanks that in many subjects produced acute psychological reactions and vivid recurring fantasies and most subjects withdrew in under 8 h [37]. In the real world the sensory deprivation environment is more basic than that found in the laboratory being achieved through hooding or forcing captives to wear blanked-out goggles or masks, and ear defenders or earflaps to impair hearing.

The symptoms associated with sensory deprivation and perceptual deprivation have been well-documented and replicated across various studies [38, 39]. One of the most important results of sensory deprivation experiments is that the resultant psychologic disturbances are virtually universal and comprise a degree of cognitive impairment, auditory and visual hallucinations, increased susceptibility to suggestion, instability of belief and attitude change, increased compliance, anxiety and depression, apathy, lethargy, reduced stimulation-seeking behaviour, disorganised planning and eventually depersonalisation which causes some people to lose touch progressively with reality [40, 41].

Certain factors can moderate the amount of stress caused by sensory deprivation and increase a person’s ability to tolerate it, especially knowledge of the duration of the experience and an awareness of the passage of time [42]. Coping methods employed include talking aloud, recitation, humming, concentrated and directed thinking, problem solving and future planning.

It has been pointed out that sensory deprivation laboratories create highly artificial situations and that the comparatively large number of subjects who fail to complete an experiment differs markedly from those who successfully adapt to isolated and sensorially deprived environments that are less artificial in construction [3].

### Sensory overload

Much research has been conducted into the effects of sensory deprivation and isolation but far less into sensory overload although evidence suggests that overstimulation is more aversive than under stimulation [34]. Sensory overload, which has been an increasingly used technique in recent years, is often auditory (e.g., playing rock music incessantly over loudspeakers in prison camps), or visual (e.g., lights being kept on 24 h a day, flashing strobe lights). Other sensory overload methods can be more subtle: in 2006 a British Muslim documentary maker was arrested whilst filming in Peshawar. He was hooded and taken to an interrogation centre, ‘When I was able to see, I got a peek into other rooms. I saw two crouching men with long black beards. A guard said they were Afghans who had been there for months. One was in a cell painted with black and white spirals to drive him mad’ [43].

An early reported use of sensory overload was in 1998 when Panamanian President, Manuel Norriega, sought to avoid arrest by seeking refuge in the Papal Nunciatura (the Vatican Embassy). Originally intended to restrict his internal and external communications loud rock and hard metal music were played through loudspeakers surrounding the Nunciatura. Norriega surrendered after 10 days. The effective component is not music but sound and it appears that sound irritation works especially well in urban settings as the sounds repeatedly reverberate off the walls which disorientates and confuses the enemy [44].

It is not clear when sound was used specifically against individuals, however, it was reported that ‘uncooperative Iraqis’ were being exposed to music such as Metallica and even Barney the purple dinosaur from a US children’s television show. The tactic was designed to reduce a captive’s resistance through sleep deprivation, frustration and irritation particularly with music that was culturally offensive. According to one proponent, ‘These people haven’t heard heavy metal before. They can’t take it. If you play it for 24 h, your brain and body functions start to slide, your train of thought slows down and your will is broken’ [45]. Another reported that detainees subjected to strobe lights and loud rock and rap music for up to 14 h a day became, ‘…very wobbly. They came back to their cells and were just completely out of it’ [46].

The captive has no control over any sensory attack; he or she cannot predict or control its output; cannot withdraw or evade from it or habituate to it, the person cannot screen the incoming stimuli and this can overwhelm their psychological defence mechanisms. Although habituation to noise can occur in healthy subjects, sounds of sufficient intensity, significance, duration or stimuli that imply conflict do not completely habituate [47].

Experimental studies with subjects exposed to intense auditory and visual stimuli showed heightened and sustained arousal, discomfort, mood changes, illusions and hallucinations and body image distortions, irritability, distraction, disorientation and a withdrawal from reality. Early work in this area reported that sensory overload could produce symptoms similar to various pathologies and produced thinking and behaviour, particularly speech content, associated with schizophrenia [48].

Sound irritation does not need to be loud. The use of ‘white noise’, which is perceived as a continuous background hiss, is used to overstimulate and irritate a captive and to disrupt cognitive processing. Noise below 80 dB can impair task performance particularly on complex, multi-component tasks that involve attentional processing [49], and there is some evidence to suggest that noise interferes particularly with information integration [50].

Noise in exceptional environments can become a stressor, e.g., in ICUs continual meaningless noise from equipment, staff activities, conversations etc. can be confusing, irritating and at times loud [51]. It is also the case that we prefer natural sounds rather than ones produced artificially in the environment; the sounds of the countryside rather than those of a townscape are more congenial and this preference has been suggested to relate to the fact that artificial soundscapes have more aperiodic sounds whereas the human brain prefers more harmonic and periodic sounds [52]. Sounds that are perceived as aperiodic tend to be perceived as unpleasant and interestingly, the most threatening sounds from mammalian predators show aperiodic spectra that are perceived as being harsh [53].

### Sleep deprivation

Sleep disturbance can occur in normal environments and has been regularly reported in exceptional environments such as space missions, polar expeditions, ICUs and so on [24, 54]. In space sleep tends to be shorter, more disturbed and shallower than on Earth [54]. Deliberate sleep deprivation can also occur in exceptional situations (e.g., single-handed yacht cruising, military operations etc.) as well as under torturous conditions. Sleep deprivation and sleep disruption were used at Guantanamo Bay detention camp under a policy officially approved by the US Secretary of Defense (April, 2003). Known in-house as the ‘frequent flyer programme’, but more officially as ‘sleep adjustment’, its aim was to lessen a detainee’s resistance and to disrupt the formation of cell relationships [55].

One recently documented case is that of Mohammed al Qahtani who has been held at Guantanamo since 2002 and interrogated under a protocol known as the ‘First Special Interrogation Plan’ [56]. He was permitted to sleep for only four or fewer hours at a time followed by interrogation sessions lasting up to 20 h. His rest periods were disrupted by constant lighting, loud noise, being moved to different cells and by allocating sleep periods during the day, a technique known as ‘sleep cycle inversion’. If he began falling asleep during interrogations he would be forced to stand or sit and water would be poured over him. Al Qahtani reported visual and auditory hallucinations that were consistent with the effects of sleep deprivation. A study into the torture of 50 individuals in Iran since 2009 identified twelve people who described being subjected to tactics designed to deprive them of adequate sleep. Guards would bang repeatedly on their cell doors, water was thrown on them or, as soon as they fell asleep, they were woken and taken for interrogation. Excessive stimulation included having a very bright light constantly on in their cells and having religious tracts broadcast at loud volume for long periods [57].

During the 1970s the British government admitted to using sleep deprivation as a technique in conditioning suspected terrorists in Northern Ireland prior to interrogation. Sleep deprivation as a deliberate technique is no longer used by the British following a complaint to the European Court of Human Rights. The Court determined that, whilst sleep deprivation did not constitute torture, it did constitute inhumane and degrading treatment [58]. The United States still retains sleep deprivation (‘adjustment’) as an official conditioning technique in the interrogation arsenal of the Central Intelligence Agency (CIA) although apparently it is not used by the Federal Bureau of Investigation.

Sleep as a behavioural activity is not fully understood, but at a basic level it is essential for survival and to support mental and physical health. Sleep also enhances immune system function and conversely lack of sleep degrades immune function [59]. There are three general categories of sleep deprivation: Long-term total sleep deprivation (>45 h), short-term total sleep deprivation (≤45 h) and partial sleep deprivation (<7 h in a 24 h period) [60].

Sleep restricted to between 3 and 6 h decreases working memory performance and increases attentional dysfunction [61]. Sleep deprivation in excess of 16 h produces deficits in attention and executive function, impaired language skills and communication, reduction in working memory capacity that is needed to process on-line information from the environment, loss of situational awareness, over-reliance on previous strategies, unwillingness to try out novel strategies and unreliable memory for episodic events [62, 63].

Four or more days of partial sleep restriction results in cumulative adverse effects on neurobehavioural functions [61] including alterations in language processing with problems in both the transmission and receiving of messages [64, 65] loss of vocal intensity, increasing length of pauses, poor enunciation, slurring and mumbled instructions. Two weeks of sleep restriction (4 h per night) produced deficits in attention, working memory and cognitive function equivalent to that found after two nights of total sleep deprivation whilst 2 weeks restricted to 6 h sleep per night produced cognitive dysfunction equivalent to one night of total sleep deprivation. Eventually personality and rational behaviour begin to disintegrate leading to apparent psychosis and mania; perception becomes disorganised and potent hallucinations are common sometimes accompanied by paranoia.

Sleep deprivation impairs performance not only through a simple want of rest but also through the disruption of a person’s diurnal rhythms. This condition is known to occur in polar regions producing sleep disturbances during the period of darkness with a decline in feelings of well-being and alertness and similarly in space due to loss of the 24 h light/dark cycle [3]. Evidence suggests that the body possesses at least two endogenous biological ‘clocks’ that govern waking behaviour through modulating core body temperature and various endocrine functions and difficulties arise when these are thrown out of phase, a condition known as circadian desynchrony [66]. Internal clocks are set or reset by external cues known as *zeitgebers*, which may be physical (e.g., the light/dark cycle of night and day) or social (e.g., mealtimes, clock-times, etc.). These cues will act upon the biological clocks either to bring the body cycle into adjustment or conversely to discourage readjustment. The evidence so far suggests that changes in environmental, physical or social conditions that affect these diurnal rhythms will produce sleep disturbance. Similarly, sleep disturbance can disrupt diurnal rhythms.

Given the rapid cognitive disorganisation that occurs through enforced sleep loss and the quick onset of paranoid symptoms, hallucinations and loss of personal control, it is perhaps not surprising that sleep deprivation has been a favoured tool for reducing resistance in non-compliant captives. To resist coercion the captive has to be able to think cogently and to monitor his or her own condition, but the psychological process most prone to disorganisation by lack of sleep is self-regulation and we are notoriously inept at monitoring our own condition. This is the reason that sleep deprivation is effective in reducing a person’s ability to resist interrogation.

As an historical aside, sleep deprivation was a favoured technique of Matthew Hopkins (c.1620–1647) the so-called ‘Witchfinder General’ who operated around the counties of South-East England interrogating and causing to be executed approximately 230 putative witches. In England at that time torture was illegal even by the Church; however, sleep deprivation was not considered to be torture. His first victim was kept without sleep for three consecutive nights but succumbed and confessed to being a witch during her fourth sleepless night incriminating five other women in the process. On another occasion John Lowes, the rather unpopular minister of Brandeston, was accused of being a witch. He strongly denied his guilt but again he was deprived of sleep for several days and nights until, ‘…he was weary of his life and scarce sensible of what he said or did’, and consequently confessed to having covenanted with the Devil. When he had recovered his sleep he retracted his confession but was hanged anyway [67].

Whilst a person may be subject to total sleep deprivation the more common problem is that of chronic partial sleep restriction, in which a person fails to obtain sufficient sleep to maintain healthy cognitive function. It has been claimed that cognitive function is affected more by partial than by total sleep deprivation [68] producing impairment particularly in attention, working memory and cognitive fluidity [61]. At least 4 h sleep is deemed necessary to maintain executive type skills that include higher order thinking and planning [63].

Not all cognitive functions are affected equally, and some complex higher order cognitive functions remain quite robust [63]. Those tasks that involve convergent skills (e.g., logic-based thinking) seem to be resistant to the effects of sleep deprivation whereas divergent tasks involving flexible reasoning and multitasking are particularly prone to sleep deprivation such as working memory processing, assimilating changes in on-line information, memory consolidation, updating strategies based on new information, lateral thinking, innovation, risk assessment, monitoring outcomes, mood-appropriate behaviour (involving self-regulation), insight, communication and temporal memory skills [69].

Performance that is based on previous training and rule-based cognitive procedures seems more resistant to sleep deprivation, however, impairment in innovative, novel and flexible thinking can lead to perseveration of action and thought; in other words, previously learned training becomes the only option available even if it now ceases to be adaptive in a new and extreme environment. Lack of sleep also produces an element of confusion that is indicative of executive dysfunction. Recovery from sleep loss does not occur overnight and tends to require a minimum of 13 h sleep for initial recovery no matter how long the person has been sleep deprived [70].

### Temporal disorientation

As well as the physical environment the temporal environment can also be manipulated. In normal circumstances it is possible to predict events with a degree of certainty: getting up and going to bed; meal times; work and play activities etc. Along with the ability to predict such events a person also has some control over them and their timings. He or she can retire to bed earlier or later, engage in or disengage from a work or play activity and so on. However, it becomes stressful when the ability to control or predict near term events is lost. Denying people the means of telling the time or even knowing day from night is a common practice designed to cause confusion and cognitive disorientation. This can be achieved through removal of watches and other timepieces, manipulation of clocks, sleep inversion, exclusion of natural light, broken shift patterns, allocating pseudo-random times for meals, showers and otherwise regular activities.

An Australian national, who was convicted for heroin smuggling in Indonesia and executed in 2015, described his final prison existence as being, ‘…like some sort of limbo or purgatory before we are punished […] Here, for some reason, they won’t allow us to know the time which is weird and can be a little disorienting […] The isolation is tough, its maddening not knowing what’s going on in the outside. Before I was so connected with what’s going on—I felt a little in control whether the news was good, bad or ugly. Here I feel completely helpless’ [71].

Most people have a low tolerance for confusion and ambiguity. Consequently, manipulation of near-term events in a person’s life can be used to increase pressure by creating confusion that utilises extra resources within the supervisory system that would otherwise be used for self-regulation thus reducing the ability to resist. Psychogenic shock produces an initial acute confusion that disrupts self-regulation and cognitive processing [72]. This confusion can be extended by making sudden changes in routine, surprise moves to a new detention facility, and allocating new guards and interrogators. These tactics can increase ambiguity and confusion in a person that decreases further his ability at self-regulation. The loss of control over near-term events can lead to regression or the adoption of a childlike state of dependence.

## Psychophysiological

### Stress positions

There are two types of positional torture: one involves being ‘stressed’ such as having wrists bound and being suspended from hooks in the ceiling; the other involves ‘stress’ in which a position is assumed and has to be held for a long time, such as ‘wall standing’ in which the captive is forced to lean against a wall at around a 45° angle, with arms outstretched, sometimes on fingertips, with feet back and spread apart. An alternative is the ‘ski’ position in which the captive squats with his back against a wall, thighs parallel to the ground and arms outstretched. The victim is forced to maintain this position for extended periods of time and often for hours. Stress positions do not have to be quite so artificial as simply standing to attention for long periods induces pain and fatigue.

One report on torture in Iran describes stressed positions including having wrists bound together behind the body (‘reverse suspension’ or ‘strappado’) and suspended with the toes either just touching the ground or just clear of the ground; knees bent and hands cuffed under and behind the knees with an iron bar inserted through the back of the knee and crook of the elbows and then suspended. Stress positions were also described such as being stretched out face down on the floor with arms extended and the body weight taken on the fingertips; standing on one leg holding cuffed hands above the head over a prolonged period. Sometimes both forms of positional torture would be combined, for example, standing with hands cuffed above the head and with weights hung from the scrotum; being confined inside a small container over a prolonged period; lying face down with wrists bound to the front legs and ankles [57]. The former can be considered physical torture whereas the latter has a significant psychological element because it is the captive’s own body forced against itself that is causing the pain.

The important factor is not the pain itself but the person’s perception of that pain. An individual readily distinguishes between aversive or painful stimuli that occur naturally (e.g., rheumatism), are inflicted by others (e.g., corporal punishment) and those inflicted by himself (e.g., through competitive sport). The person’s response to the effect of the pain is determined by his perception of the cause of the pain. Stress positions can encourage an individual to see himself as the cause of his own pain.

Stress positions can lead to long term or permanent damage to nerve, joint and the circulatory system, causing chronic pain and restriction in movement. Blood vessels, especially arteries, need physical movement to function well. If this movement is prevented, through prolonged standing, for example, then pooling of the blood in arterial extremities, tissue swelling, numbness and the formation of blood clots can occur. Reduced blood supply to the brain, particularly to the temporal and prefrontal cortices, produces impairment in executive functions [12].

### Thermal stress

It is not uncommon for people to be subjected to thermal stress (heat or cold) when working in polar regions, deserts or jungles, underground in mines or under water. In these situations attempts are made to adapt or to reduce the intensity of the thermal strain through clothing, heaters, air conditioning units etc. Equally, thermal stress can also be used deliberately against people held in an environment that is either too hot or too cold for personal comfort. This situation may arise by default, e.g., being held captive in the Middle East is likely to lead to heat stress especially if the person is held in a confined space. It is also known for temperatures to be adjusted deliberately to undermine a person’s motivation to resist and to impair cognitive function. During World War II the German interrogation facility (Auswertestelle West) contained 200 cells in which the PoWs would be held in solitary confinement for up to 5 days, although 30 days holding was known. These cells had electric heaters that could make the temperature insufferably hot. In 1945 three German personnel were sentenced to terms of imprisonment for mistreatment of British PoWs by this method. In Iraq a bodyguard of Saddam Hussein was captured and reported that he was forced to strip and placed in front of an air-conditioner whilst cold water was poured over him [73]. Similar practices occur elsewhere with reports of cold water being poured over a person who was then left outside in winter so that his clothes froze on his body [7].

Thermal effects on physical and physiological performance are well documented. Thermal stress on cognitive function, particularly higher order cognitive function, is less well established and the results are mixed [74]. Performance on perceptual-motor tasks is perhaps the largest domain in which thermal stressors have been examined. Studies into vigilance tasks in heat and more manual functions in cold reflect the pragmatic purpose of much of the original empirical research primarily focussing on particular work environments and military settings [75].

Cold decreases strength and endurance, and impairs tactile sensitivity at 8–10 °C; manual dexterity, fine motor movements, hand strength and small object manipulation at 12–15 °C [75, 76]. There are few studies that have examined directly the effects of cold on cognitive performance but the findings available suggest that tests which require relatively minimal cognitive processing are unaffected by either initial cold stress or by later central cooling whereas tasks requiring more higher-order cognitive processing seem to show a slight improvement upon initial cold (possibly due to increased arousal) but a significant decrement following later central cooling [77]. Working memory and attentional encoding processes (learning) are impaired with significant cold although long-term memory and the recall of previously learned information remains unimpaired [78].

Vigilance and sustained attention tasks are the most commonly tested under heat exposure. The overall pattern of effects for heat is somewhat confusing and appears to depend on the task examined and the intensity of the heat experienced. Moreover, when the temperature remains constant (albeit hot), performance decrement is much less than when the temperature is variable or climbing.

The overall findings are not straightforward: mild heat exposure appears to impair performance on vigilance tasks, while further heat stress may improve performance. For more complex mental tasks involving working memory and higher information processing, the effect is reversed, initial facilitation followed by impairment. Furthermore, these effects may vary with the duration and severity of exposure and the characteristics of both the task and the individual [76, 79].

A meta-analysis of thermal stress effects (comparing heat and cold) indicates that whilst heat does not significantly affect the speed of performance it can degrade accuracy and cold significantly impairs both speed and accuracy [80]. The more cognitively demanding the task, the more it is prone to impairment with either heat or cold [75].

## Psychosocial

### Cultural stress

Problems can arise in exceptional environments through cultural stressors, usually as a consequence of mixing people together from different cultural backgrounds, although attempts are being made to reduce these effects. Differences in beliefs, behaviour and cultural backgrounds can increase interpersonal tensions and miscommunication with the result that teamwork and the establishing of operationally functional relationships can be impaired [81]. Language and cultural factors were found to isolate minority crew members during space missions, simulated isolation studies and polar research stations [3].

Psychological torture includes the manipulation of psychosocial factors to attack a person’s religious, social, professional or personal mores. These behavioural attacks can involve the removal of religious artefacts as well as the denial of prayer and other religious observances; the forcible shaving of beards and hair with religious or societal connotations; the denial of his or her professional status, military rank or occupational standing; the witnessing of the abuse of a friend, spouse or child; being forced to engage in activities contrary to their personal, cultural or religious values; to ‘perform’ for their captors often whilst being laughed at or mocked and the swapping of their name for a number. Tactics of humiliation reported in 2014 included the forced removal of clothing, being spat on, ejaculated on and urinated on; being forced to drink urine; being watched whilst going to the toilet and being forced to dance semi-naked [82]. Such acts instil a sense of both helplessness and hopelessness. Helplessness combines depression and anxiety whilst hopelessness combines depression and guilt.

One aim of psychological torture is to, ‘…disintegrate the individual’s personality. The torturer attempts to destroy a victim’s sense of being grounded in a family and society as a human being with hopes, dreams and aspirations for the future’ [83]. A key factor here is the coercion of one person by another with a clear power differential between them that undermines the captive’s personal identity and sense of worth. The captive is debased, denigrated and degraded. With reference to Article 3 ECHR a treatment is considered ‘degrading’ if its object is to humiliate and debase the person and if the consequences adversely affect his or her personality. The philosopher Kant argued that human beings are ends-in-themselves by possessing free will, therefore, to treat a captive as a means to an end rather than an end in himself, is to humiliate him by denying his claim to humanity [84].

‘Humiliate’ was originally associated with ‘humility’ and the condition of being ‘humble’, meaning to be of lowly condition (thirteenth/fourteenth century), and ‘to humiliate’ meant to humble someone. After the sixteenth century ‘to humiliate’ referred to debasement, literally a de-gradation of a person’s status and dignity. The earliest record of humiliation meaning to mortify or to undermine the dignity or self-respect of someone occurs in 1757 [85]. Whereas humility still retains positive associations, humiliation does not. Humiliation is traumatic and the person suffers a stunned loss of dignity and of personal self-worth. It is an attack on a person’s identity that leads to a feeling of worthlessness.

It has been argued that the infliction of severe humiliation is a violent psychological act in which the person suffers an annihilation of the self that can leave the victim with residual trauma lasting many months and even years [86]. Furthermore, the trauma of severe humiliation can become internalised producing psychiatric conditions such as severe depression and anxiety with flashbacks, nightmares, sleeplessness, apathy, depression and symptoms comparable to PTSD with suicidal ideation [87]. People who have been humiliated carry the imprint of their humiliation and describe feeling that their self-identity has disintegrated by their experience, that they are less than human and unable to live as a normal person.

### Sexual degradation

Strip searches are often imposed for security reasons, to check for any concealed items or substances that the captive may have attempted to hide on his or her person. A captive may be stripped for intelligence purposes as information can be obtained from markings such as tattoos and scar tissue. However, captives may also be forced naked, often violently, for coercive purposes: e.g., to induce shame or humiliation, especially in the presence of members of the opposite sex; to undermine a sense of dignity; to emphasise the power differential between captive and captors and to increase fear of sexual and physical assault. Stripping a person of his or her clothes begins the process of stripping them of their identity and their personality, a process that saw its complete expression in the Nazi concentration camps. Sexual violations can be particularly shameful if they cross cultural taboos relating to e.g., pornography, homosexuality, women etc. or by being forced to carry out simulated or actual sexual acts [7]. Sexual assault can be applied to both men and women including rape, molestation, violence to genitals and penetration with an instrument as reported by former victims [57].

Psychological assault inflicted through sexual abuse was revealed in the photographs that emanated from the Abu Ghraib prison in late 2003. Reports detailed how prisoners suffered sexual abuse, rape and sodomy and were forced to carry out simulated sex acts. One photograph showed a group of male prisoners, stripped naked and forced to form a human pyramid. The man on top later reported that he was humiliated so much that he became despondent and suicidal [88].

Rape, meaning sexual aggression with penetration, is a physical act that also constitutes a psychologically degrading and inhuman treatment and has now been officially defined as being a form of torture [9]. For example, in a case brought before the European Court of Human Rights the applicant claimed to have been raped whilst in police custody. The Court, finding with the applicant, stated that:

‘…rape of a detainee by an official of the State must be considered to be an especially grave and abhorrent form of ill-treatment given the ease with which the offender can exploit the vulnerability and weakened resistance of his victim. Furthermore, rape leaves deep psychological scars on the victim that do not respond to the passage of time as quickly as other forms of physical and mental violence. The applicant also experienced the acute physical pain of forced penetration, which must have left her feeling debased and violated both physically and emotionally’.

The Court found that this rape amounted to torture in breach of Article 3 of the European Convention on Human Rights [5].

Sometimes deep psychological scars are inflicted deliberately through mass rape to cause shame, intimidation, degradation and humiliation on a targeted population often with the aim of forcing a particular group of people to leave a geographic region. A modern example is the mass rape carried out by Bosnian Serb forces against 20,000–50,000 Croatian and Bosniak (Muslim) women during the 1992–1995 war which was used as a strategic weapon of ethnic cleansing. A medical study examining the psychological consequences of 68 victims of this rape by Serbian forces found that many suffered psychological problems as a result although none had any reported psychiatric history prior to the rapes [89].

Specific psychological responses to sexual torture include: depression and anxiety; intense and overwhelming feelings of shame; involuntary and intrusive flashbacks of both the events and the perpetrators; feelings of anger towards the abusers; fear and severe anxiety symptoms; avoidance of anything associated with the trauma, including amnesia for details of the trauma; social withdrawal and difficulty making relationships; sexual dysfunction; depersonalisation, dissociative states and in severe cases suicidal ideation and suicide attempts [57, 82].

Sexual duress can also occur within exceptional environments often because there is difficulty in evading or avoiding it. In one space station simulation study it is reported that tension developed as a result of unwanted sexual advances of one male crew member to the lone female crew member which may also have been related to cultural differences as both were of different nationalities [90].

## Cluster effect

Whilst individual forms of duress (isolation, sensory and social deprivation, thermal stress, noise etc.) can have a psychological impact it is the clustering of these environmental stressors that appears to be particularly pernicious producing psychological impairment in exceptional, extreme and tortuous environments; in fact, the polar winter over syndrome, and accompanying impaired cognitive function, has been attributed to such a cluster effect [91].

Whilst each psychological stressor may produce distress individually they may not be deemed ‘torturous’, e.g., a former president of the American Psychological Association when asked by CIA psychologists whether sleep deprivation constituted torture, concluded that sleep deprivation is *not torture on its own* [26; my italics]. However, taken together the combination of sensory assaults produces a cluster effect that can be particularly damaging. Deliberate clustering of psychological assaults are reported to have been used in Afghanistan including enforced nakedness, isolation for long periods, stress positions and sleep and light deprivation. Similarly, the International Committee of the Red Cross reported combinations of psychological assaults on detainees in Iraq including threats, insults, verbal abuse, hooding, sleep deprivation, forced nudity and sexual humiliation [92]. It has also been argued that, in cases of torture, three different processes (e.g., beating whilst blindfolded and with hands tied) constitute three different stressors the effects of which are multiplicative not additive [93]. Consequently, the distress associated with each event is largely determined by the interactional impact of all three events which can be further aggravated by removing from the victim any control over the process.

This cluster effect has been recognised by the United Nations, which states that cumulative effects should be taken into account to determine whether a case amounts to torture [7]. A similar acknowledgment of such a cluster effect has been recognised by the European Commission of Human Rights which determined that five methods of conditioning suspected terrorists by the UK government in the 1970s (standing spread-eagled against a wall, hooding, ‘white noise’, sleep, food and drink deprivation) did not ‘rise to the level of torture’. This was later disputed and it was ruled that the five methods used together did constitute ‘inhuman and degrading treatment’ with both the European Commission and European Court ruling that the cumulative effect had to be taken into account and not only each component separately [58]. The concern is less to do with a haphazard collection of techniques that may amount to ill-treatment, but rather that all these methods combined can form a system designed deliberately to undermine an individual, to disrupt the senses and to disintegrate personality. The effect over a prolonged period of time of this clustering has to be considered a part of psychological torture [9].

## Pathogenesis vs. salutogenesis

It is not surprising that undergoing psychological duress can have psychological repercussions that may be pathogenic producing some degree of psychological debility. Following tortuous experiences persistent symptoms may include incoherent speech, disorientation, hallucination, irritability, anger, delusions and sometimes paranoia [46]. Post-assault symptoms include impairment in cognitive function particularly memory, attention and concentration; somatic complaints such as headache and back pain, hyperarousal, avoidance and irritability. There may also exist severe depression, apathy and feelings of shame and humiliation [94]. Following cases of actual torture, victims may present with post-traumatic stress disorder (PTSD) although this is not always straightforward. Some consider that the victims of psychological torture suffer a more complex PTSD or ‘extreme stress disorder’ and others have argued for the existence of a specific ‘torture syndrome’ characterised by impairment in cognitive function, particularly memory and concentration, sleep disturbance and nightmares, emotional lability, anxiety, depression, and somatic complaints [95]. A ‘dose effect’ of traumatic exposure with a near linear result of traumatic events has been identified with a proportion of PTSD [96]. Furthermore, stressor clusters, rather than individual stressors, seem to relate to PTSD as they reflect better the cumulative impact of stressor events [93]. Interestingly, in one study of 432 torture survivors PTSD was found to be related to captivity, deprivation, sexual torture, exposure to extreme temperatures, isolation and forced stress positions but not to physical torture and that many survivors did not develop PTSD despite severe torture [93].

Not everyone is severely affected by experiencing extremis and different people can react differently to the same environment, even to an extreme environment; consequently, it is not the environment *per se* but the meaning that people attach to their experiences in that environment that is the determining factor [97]. One Briton who survived being held hostage in the Middle East for nearly 2 years interpreted it thus, ‘I saw it as an interesting cultural experience, but not one I wish to repeat’ (personal communication).

One interesting theme that is emerging from studies of people in differing environments is the positive or salutogenic effects of undergoing such experiences, usually by the successful application of strategies to cope with the adversities. Positive effects have been noted in exceptional, extreme and tortuous environments including submarines, polar research stations [1, 4, 22, 98], space missions [23], and even amongst survivors of genocide and persecution including the Holocaust [99].

## Conclusions

Our cognitive system has adapted to support goal-directed behaviour within our normal environment over which we have some degree of control. Some people will choose to live and work in an exceptional environment, such as a polar base, space station, submarine etc.; an environment to which they are not optimally adapted but can find some degree of accommodation usually through the development of coping strategies. Other exceptional environments also exist, but these are not voluntarily chosen, such as prisons, ICUs, isolation wards etc. and which also require coping skills for optimal adaptation. An extreme environment is marked not only by a more intense environmental experience but also by a real or perceived lack of control over the situation such as occurs in surviving at sea in a life-raft or in a harsh prison camp. The experience of an extreme environment can become tortuous when specific environmental stimuli are used deliberately against a person usually in an attempt to undermine his will or resistance.

The main coercive environmental factors comprise three categories: psychological (isolation, sensory deprivation, sensory overload, sleep deprivation, temporal disorientation); psychophysiological (thermal, stress positions), and psychosocial (cultural humiliation, sexual degradation). Each factor on its own may not be considered tortuous, however, if deliberately structured into a systemic cluster may constitute torture under legal definition [9].

The pathogenic effects of suffering extreme stress have been well documented (although whether a specific psychiatric syndrome exists is still debated) and the evidence suggests that the cognitive processes most vulnerable to psychological assault, especially when combined with the self-inflicted pain from stress positions, is executive function. This results in intellectual deterioration, difficulty in focusing, sustaining attention and psychological balance; in severe cases tortuous techniques can compromise the integrity of the mind–body system causing disintegration of a person’s identity and personality which may lead to regression or psychiatric disorder.

The premise that an environmental spectrum exists that passes from optimal, through exceptional, extreme to tortuous provides a framework to enable a better interaction to be found between a person, the environment in which he or she has to work and the task that has to be undertaken, even if that task is survival itself. The more salutogenic, or health giving benefits, of undergoing exceptional, extreme and even tortuous experiences should be recognised further and ways to capitalise on these positive experiences, as well attempting to ameliorate the more unpleasant aspects of an abnormal environment, should be sought.

## Abbreviations

CIA: Central Intelligence Agency
ECHR: European Court of Human Rights
ICU: intensive care unit
PTSD: post-traumatic stress disorder
UN: United Nations
